# Evolution and Features of China’s Central Government Funding System for Basic Research

**DOI:** 10.3389/frma.2021.751497

**Published:** 2021-12-23

**Authors:** Aruhan Bai, Cong Wu, Kejia Yang

**Affiliations:** ^1^ Institutes of Science and Development, Chinese Academy of Sciences (CAS), Beijing, China; ^2^ University of Chinese Academy of Sciences, Beijing, China; ^3^ Science Policy Research Unit, University of Sussex Business School, Brighton, United Kingdom

**Keywords:** basic research, central government, funding system, China, historical evolution

## Abstract

Basic research is believed to be a crucial factor for building national innovation capacity and therefore was perceived as a key battleground for national technological and economic competition. Since the economic reform and opening up in the late 1970s, China has made great achievements in building up its national research system. However, the lacking capabilities to conduct ground-breaking scientific work remain one of the daunting challenges for the country. How to restructure its funding system for basic research so to reinvigorate its indigenous innovation capacity has been one of the main concerns for the Chinese government in recent years. To address this, the paper proposes a conceptual framework to analyze how China’s central government funding system for basic research has evolved since 1985. The paper concludes with a discussion of the identified problems and challenges that China is facing in its current funding system for basic research.

## Introduction

The world today is undergoing major disruptions in its geopolitical order under the impact of multiple factors such as the changing US-China relationships and the ongoing COVID-19 pandemic. Science and technology competitions have been a key focal point in this shifting new global order. Under this context, basic research was perceived as the core battleground for the state investment competition because of its crucial role in building national innovation capability.

China, now a major performer in the global research system, has made great achievements in levelling up its research capacity over the past few decades (Marginson, 2021). This has been evidenced in its rapidly increasing investment in R&D, the great expansion in the numbers of research personnel and publications. According to the data from OECD^1^, China’s investment in R&D has witnessed a growth from 15.95 billion (Chinese yuan) in 1991 to 2.214 trillion (Chinese yuan) in 2019; the numbers of researchers increased from 3.18 million (1991) to 7.12 million (2019); and according to the statistics from the US National Science Foundation (NSF), China reached No. 1 in the world by 528,263 publications in the S&E field in 2018^2^. However, China is still in the process of catching up and a transition period of transforming itself into an innovation-driven development country (Liu et al., 2017). Especially in recent years, China suffers from the external technological stranglehold imposed by Washington, evidenced in its semi-conductor sector which has severely undermined its industry development under US sanctions. To fundamentally address this issue, improving its research capacity and indigenous innovation capability has been perceived as a solution. However, it remains one of the persistent challenges for the country to level up its original innovation capability (*yuanshi chuangxin*), i.e., the capability to conduct ground-breaking scientific work, such as the scientific breakthrough that can lead to a Nobel Prize. To fundamentally alter the status quo, the central government is urged to adopt measures to include increasing the investment in basic research and restructuring its funding systems, so that to strengthen its research capacity in responding to the country’s strategic needs through leveraging what has been labelled as “strategic basic research” (Fang, 2019).

There are a wide range of policy measures that can be adopted for latecomers to narrow their research gap to the frontier countries. One of the common measures is to level up the national research capacity through directly increasing public investment in R&D activities. This was believed to be able to build up the country’s absorptive capacity which enables itself to fully leverage the knowledge transferred from other leading countries. In this sense, to bring the full potential of the central government’s funding for basic research, it requires horizontal coordination with other policy support, such as setting up policies to attract the talents around the world, or to attract the R&D intensive Foreign Direct Investment (FDI). Moreover, it is widely acknowledged that apart from directly publicly supporting research related to national priorities, the public investment into R&D should also produce a domino effect in resource commitment, inducing the private investment into R&D (Wallsten, 2000). The public and the private sector can build joint efforts to co-fund research institutes (e.g., nanoelectronics lab *imec* in Belgium).

This paper focuses on the Chinese central government’s funding for basic research as it has been the main investor for basic research in the country and will continue to be so in the anticipated future. To be specific, we address the following research questions: how have China’s central government funding systems for basic research evolved? What are the problems and challenges it is facing today? And what are the opportunities to improve its funding system in order to build more efficient research systems in responding to its new international challenges and domestic development needs?

Most of the existing studies on China’s basic research focus on the performance evaluation based on input and output or its resource allocation structures (Cao, 2020; Xia et al., 2020). Zhu and Gong (2008) suggested that China invest less in basic research than the world’s leading innovative countries, such as France and the US. Huang et al. (2015) found that before 2010 the country paid limited policy attention to basic research compared with applied research. Sun and Cao (2014) explained China’s R&D spending structure and introduced its major research funding agencies. Some problems are identified, for example, Yang (2016) recognized a heavy imbalanced funding allocation among universities from the National Natural Science Foundation of China (NSFC), and the lack of confidentiality and transparency in the process of making decisions for grant allocation is regarded as a key issue (Qiu, 2014).

However, there are very limited insights on how China’s central government funding system for basic research has evolved. To offer suggestions for its future development, it is crucial to understand its historical context. In this paper, we will review the historical evolution of China’s central government’s funding system for basic research since 1985 when it embarked on the reform of its science and technology system. The study is based on the rich historical data that we have collected from policy documents, national and provincial science and technology statistics, organizational reports (such as China’s Basic Research Competitiveness Report), and archives such as the news articles and research reports (see the Supplementary Appendix Table S1 for detailed data sources).

The paper is structured as follows. *Funding for Basic Research* first reviews the nature of basic research, and then it identifies the central government funding models for basic research. *Analysis Framework of Funding System for Basic Research* introduces our analysis framework for the funding system for basic research. *Evolution of China’s Central Government Funding System for Basic Research* presents a historical review of Chinese central government funding for basic research since 1985, and *Features of China’s Central Government Funding System for Basic Research* discusses its changing features. The final section concludes with the identified problems and challenges faced in China’s current state funding system for basic research.

## Funding for Basic Research

### The Nature of Basic Research

Basic research is generally defined as research activities “performed without thought of practical ends. It results in general knowledge and an understanding of nature and its laws” (Bush, 2020, page 17). The curiosity and desire to understand and explore the unknown are recognized as the key driving force for this kind of scientific activity. It has been widely accepted that basic research is experimental or theoretical in nature without recognizing its immediate utility (Matsuo et al., 2002).

Beyond the pure curiosity-driven “blue sky research,” Stokes (1997) argued that basic research might have clear practical implications and ideas for application. This has featured heavily in the recent argued missions or demands driven basic research (Mowery, 2009). Pasteur’s quadrant classification indicates a more complex relationship between basic and applied research, thus offering a new perspective to investigate how the state should fund basic research, whether purely support the scientists’ purpose and interests, or to select certain national needs or demands as prioritized areas. This debate raises the issue of how we should fund basic research.

Under the legitimacy of the widely distributed linear model in Bush’s report, basic research was then recognized as the source of applied research and experimental development, and it shall be taken in the scope of government funding for both economic and social benefits. Economists argue that basic research is featured as a public good which suffers from market failure (Nelson, 1959; Arrow, 1962). Basic research has been described as non-rivalrous and non-excludable. For such a good, it neither reduces the availability to others nor increases the marginal cost of subsequent users. Furthermore, due to the high cost of confidentiality, it is difficult to bar other users from the access, which may give rise to the free-rider problem. Moreover, basic research shows great positive externalities that can produce huge social benefits. Therefore, public investment into basic research has been regarded as a key solution for market failure. This theoretical rational has been echoed in the seminal report *Science––The Endless Frontier* by Vannevar Bush, published in 1945, which has provided widely accepted legitimacy of government funding for basic research.

Furthermore, basic research, as an exploration of unknown fields with endless frontiers, is full of high uncertainties and high risks, thus the government is anticipated to offer stable funding to researchers, to make sure that they can continuously conduct research to build their research continuity for scientific breakthroughs. Moreover, to guarantee the funding flowing to excellent science, it is crucial for funders to secure the freedom of researchers to conduct research aligning with their research interests and to explore new frontiers. The US NSF considers the freedom for researchers to pursue their research goals as one of the distinctive characteristics between basic and applied research (NSF, 2015). However, under accountability pressure, the government is in a rush to seek fruitful results from basic research or to channel their investment into certain priorities that are highly demanded by the country. Therefore, how to balance the two to build a more efficient national funding system for basic research has been one of the huge challenges for countries across the world.

### Funding Models for Basic Research

**FIGURE 1:**
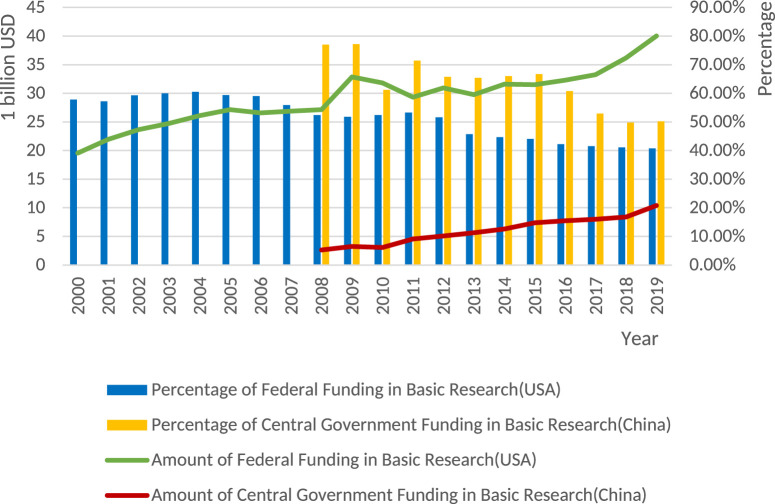
Historical data of US Federal Government and China’s Central Government Funding for Basic Research^3^. Source: Authors’ own, calculated based on the statistical data from Federal Funds for Research and Development (US NSF) and Ministry of Finance (China). Note: China’s investment has been exchanged into US dollars based on the exchange rate: 1 USD = 6.46 RMB.

This paper specifically delves into the basic research funded by the government, which has been the major supporter in most countries. As indicated in Figure 1, in 2018, the US federal government took up 42% of the national total investment in basic research, business sector accounting for 29%, universities for 14%, and others for 16%. While in China, in 2019, the central government invested 50.25% of the national total investment in basic research. Compared to the dominant role of central government, in 2020, local government only contributed to around 30% in the country’s public investment in basic research. However, it is noteworthy that the share of basic research funded by the US federal government has been declining in the past 20 years and this was also the case in China in the past decade, though the absolute amount has been going up because of the rapid increase in the total amount of R&D investment.[Fn fn3]

In terms of funding recipients of public investment in basic research, the vast majority of China’s central government funding for basic research has been directed to national research institutes and universities. Take China’s major basic research funding agency NSFC as an example, in its 2019 funded Major Programs, research institutes received 33.47% of the total fund while universities received 66.36%, and the rest 0.17% went to other sectors^4^.

## Analysis Framework of Funding System for Basic Research

**FIGURE 2:**
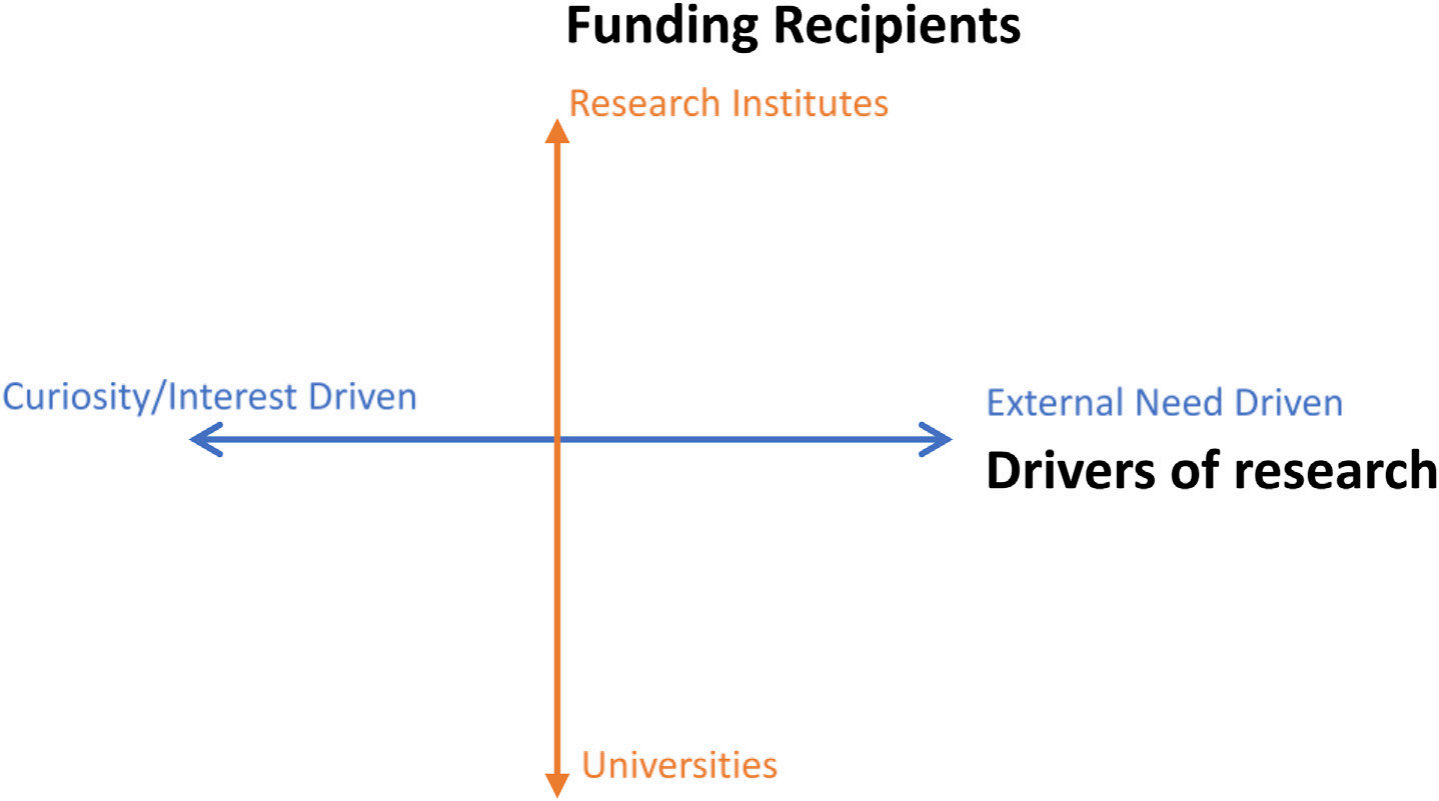
Analytical framework of funding models for basic research.

We propose two dimensions to scrutinize the evolution of funding models for basic research (represented in Figure 2). These two are: (1) drivers of basic research and (2) funding recipients.

As we have discussed, “curiosity/interest-driven basic research” and “external need driven basic research” are two typical driving models for basic research. Curiosity/interest-driven basic research derives from researchers’ personal interests, with no application purpose, represented by Bohr’s atomic theory (Stokes, 1997). The funded research is generally identified by the scientific community through peer review, with little involvement of external agencies, i.e., governments or enterprises. While external need driven research (in accord with Pasteur Quadrant) is conducted to address practical problems in social and economic development or to serve the national strategic needs at the macro level. The sources of this type of research are usually co-suggested by a variety of stakeholders, such as the government, industry sectors, and the scientific community. However, it is worth noting that there is no clear boundary between these two types of research. In recent years, it has been increasingly argued for basic science to play a role in boosting the national research capacity for social and economic development purposes, and scientists are pushed to choose research topics that are closely aligned with national priorities.

Universities and public research institutes are two major recipients of government basic research funding. On the one hand, basic research in universities is generally organized by a small research team which is represented by individual scientists, graduates, PhD candidates, and research assistants. This type of research generally holds a certain degree of freedom which allows researchers to pursue their individual research interests. The principal investigator takes responsibility which goes beyond just conducting academic research but also training students in the meanwhile. On the other hand, basic research conducted in research institutes is generally organized by larger research groups with more than one leading principal investigator. These teams typically perform as coordinated groups to complete certain research tasks which are either aligned with the missions of their institutes or aligned with national needs or priorities. Limited freedom has been guaranteed to the individual researcher within these groups. However, in China, there is a trend of homogeneity of national research institutes and universities in regard to their nature of research activities–both conduct basic research and compete to get funds from NSFC (Dai and A, 2016).

Although it has been acclaimed, in the traditional history and sociology of science, that individual genius plays a crucial role in scientific discovery (Bowler and Morus, 2010; Merton, 1968), science is increasingly being done in larger teams, which is commonly attributed to factors like the increasing “burden of knowledge” and easier long-distance communication (Wuchty et al., 2007; Jones et al., 2008; Jones, 2009; Agrawal et al., 2014). This occurs with the corresponding changes of the research funding system which increasingly encourages inter/trans-disciplinary research and cross-organizational collaborations.

Funding can provide researchers with the resources to pursue curiosity-driven research avenues, creating the conditions for taking more risk (Hollingsworth, 2004; Heinze et al., 2009). Holmstrom (1989) and Manso (2011) argued that incentive schemes that motivate innovation and explorative science must exhibit tolerance for failures. This is the case particularly if the funding is substantial and covers a period long enough to provide the researchers with the protective space to tackle ambitious questions. However, when the government holds limited research resources, it will be prone to channel these resources into those aligned with national demands or toward applied research or technology experimentations. This has been generally the case especially for catching up countries (Fang, 2019). Therefore, depending on the country’s development stages and political preferences, various funding schemes have been deployed by the government to create incentives for researchers to conduct different research activities.

Block grants and project-based funding are two common funding mechanisms leveraged by different countries to fund basic research. Block grant provides relatively stable financial support, which allows researchers to follow long-term research goals or research institutes to achieve long-term research tasks. Project funding targets around specific research goals usually provide funds only for a certain period of time and the funded researchers or teams are selected through competitions. Therefore, when it comes to comparison with the more stabilized block grants, project funding selection mechanism features high uncertainties for researchers as they may not get funded consistently following the same or related research avenues, but it enjoys the advantages in selecting high-performance projects and responding to certain real-time research problems. There are also other downsides of project-based funding, e.g., selection against highly novel projects (Boudreau et al., 2016) or duplicative research. Moreover, it costs serious efforts for researchers to prepare for research grants and the over-competition may squeeze their time and energy for conducting research activities (A and Li, 2014).

Generally, for the projects-based funding mechanisms, the research interests of individual researchers are more encouraged as the projects are mainly selected based on their research qualities and performances; while for the block grants, national demands and priorities are more emphasized as the projects will be selected in alignment with the national needs or the research institutes’ missions. Another difference between the two funding mechanisms is that for the project-based funding, the projects are generally funded for a certain period of time and researchers are impelled to produce research outcomes during this funded period. This may push researchers for publications or to generate certain research outcomes in a rush, and further undermine their determination or commitment to achieve or fulfil the long-term research tasks.

Based on the research results after examining 100 successful basic research cases conducted in China, Fang (2019) suggested that for the research which requires continuous investment following certain research topics demand stabilized grants; while for the researches which have clear research goals, for example, to achieve certain social, economic, or environmental goals, it is better to be supported by projects. However, in recent years, it is observed that the boundary between the two types of funding mechanisms appears to be blurred, in the sense that the idea of performance evaluation is incorporated into block funding management, which means it is unlikely for an institute to enjoy absolute stable funding with no extra conditions; while for project funding, certain stability can also be guaranteed based on the research performance. For example, China adopts the *performance-based rolling funding scheme (Gundong Zhichi)* (i.e., certain individuals or teams receive another round of funding support based on previous extraordinary performance) to increase its stability. However, in general, block grants are mainly channeled to the national research institutes, or institutionalized teams to address more general, macro, complicated issues, and the research topics are usually co-suggested by multiple stakeholders; while project funding mainly supports loosely organized research teams (led by individual PI), and their research topics mainly derive from the scientific community itself. How to leverage the two funding mechanisms to efficiently fund research and create a balance among personal interests oriented and national needs and demands oriented selection mechanisms has been a daunting challenge for the funding agencies across the world.

The above two-dimensional analytical framework aims to provide an angle to understand how the Chinese central government’s funding mechanisms for basic research change in practice, which will be introduced below.

## Evolution of China’s Central Government Funding System for Basic Research

Since the new China was founded in 1949, China built its research system basically from scratch. In 1956, with the call of “Marching Towards Sciences” (Xiang Ke Xue Jin Jun), China made its first *Long-term Plan for the Development of Science and Technology for 1956–1967* (hereinafter referred to as the 12-Year Science and Technology Development Plan), which was labelled as the country’s first visionary blueprint for science and technology development. The 12-Year Science and Technology Development Plan and the subsequent implemented Four Emergency Measures have effectively supported the research and development to achieve specific tasks or national missions, such as the *two bombs and one satellite* (Zhang and Zhang, 2019). During this period, because of the limited resources on capital and scientific personnel, China’s funding model for basic research follows the principles of “*national missions/tasks-oriented disciplines development* (Renwu Dai Xueke),” which means that certain disciplines were prioritized in aligning with national needs or missions. This model channeled limited resources into certain disciplines and nurtured major scientific achievements which are aligned with the country’s specific national goals. The major scientific achievements included Artificially Synthesis Bovine Insulin and Artemisinin, the latter gained China’s first and by now the only Nobel Prize in natural science.

After 10 years’ stagnation of science and technology development during the cultural revolution period (1966–1976), China entered a new period of development following its economic reform and opening up policy introduced in the late 1970s. Science and technology were perceived as the primary force for levelling up the country’s productivity and further toward its economic growth. However, there were only limited resources channeled into basic research. It was not until 1985 when China launched its reform in science and technology system that China’s modern research funding system was established. Therefore, in this paper, we examine how China’s central government’s funding system for basic research evolved after 1985. Based on the changing dynamics of stability and competition in its funding schemes, i.e., whether it is grant funding dominant or project funding dominant, the development of the Chinese central government’s funding for basic research can be divided into three different phases which will be introduced below.

Stage 1 (1985–1998): Rapid expansion of competitive project funding

In 1985, China launched its reform in science and technology system to fully leverage the force of science and technology development for economic growth. Since then, China gradually established its modern research funding system for science and technology development, which set a foundation for its current research funding system for basic research. However, in this stage, its primary goal is to overcome the division between scientific activities and industrial activities. Therefore, limited attention was given to basic research compared to applied research and technology demonstrations. To reinvigorate the creativity of scientists and build up a certain scale of scientific personnel, competition was leveraged as a dominant principle to select the national well-performed basic research. In 1986, following its counterpart, the US NSF, China set up the NSFC to provide funding for the full spectrum of disciplines in basic research. In 1997, the Ministry of Science and Technology launched the National Basic Research Programme (also known as the 973 Programme) to strengthen the capability of original innovation and provide scientific support for national development demand. Represented by the NSFC and the 973 Programme, China institutionalized its central government’s funding system for basic research, of which the project-based funding model was mobilized as a selection mechanism to build up the country’s research capacity.

From 1985 to 1998, the total amount of funding for basic research has been increased gradually. Taking the research projects funded by NSFC as an example, its gross grants increased from 86 million yuan (in 1985) to 1.026 billion yuan (in 1998). Almost all the increased funding for basic research from the central government was channeled through a competition-based project funding model. In comparison with the previous period when China was in its relatively weak economic development period, when only limited research resources were channeled to national research institutes and these only enabled them to make a living. Under the principles of a planning economy, there were simply no competitions played out. In contrast, this period’s competition-oriented funding mechanisms managed to select the best science to support the country’s needs to level up China’s science performance. Moreover, it boosted researchers’ productivity as the projects were funded based on their potential scientific performance.

Stage 2 (1998–2013): Growth of block grants and struggle to achieve a balance between “stability and competitiveness”

The previous stage was dominated by the project funding mechanism which has levelled up researchers’ productivity, yet it also raised the issue of lacking a long-term funding mechanism, in the sense that once the funded projects ended, the research came to an end, too. Thus, it has caused problems of discontinuity in the allocation of research resources to support research institutes or teams continuously following certain research areas for long-term research capacity. Limited stability was guaranteed for the researchers so that they could continuously follow their own research interests or for the national research institutes so that they can follow their missions without being continuously involved in the project’s competitions. To address this lacking in stability issue, in 1998, the central government started to increase the proportions of stable funding, mainly through increased block grants to major national research institutes, such as to the Chinese Academy of Sciences (CAS), aiming to encourage national research institutes to carry out research according to their own organizational missions and visions.

Specific research grants were channeled to both national research institutes and universities so as to stably fund their research activities. In 1998, block grants were increased through the Knowledge Innovation Programme (KIP) to CAS. This has been mobilized as a pilot for CAS to revitalize its human resources and redefine the research focuses of its research institutes (Suttmeier et al., 2006). In 2010, after successfully completing the KIP, such block funds were institutionalized to stably fund CAS’s research activities, of which basic research was a significant part. In 2006, the central government introduced *Fundamental Research Funds (Jiben Keyan Yewufei)* which aimed to support extraordinary researchers and teams from public research institutes and universities to conduct basic research on a stable and long-term basis (Ministry of Finance, 2006). This marked that block grant was applied to a wider range of actors. Following the fruitful experience of KIP, similar programs were introduced to the other national research institutes, such as the Chinese Academy of Social Sciences (in 2011) and the Chinese Academy of Agricultural Sciences (in 2013) to further enhance the proportion of stable funding to support their research activities.

Block grants were also set up to universities and were channeled through two aspects. One was through the *985 Project*, a project initiated by the central government in May 1985, aiming to build a group of world first-class universities. To support the *985 Project*, additional block grants were appropriated to certain high-level research universities to support their excellent research activities. The other channel was through the aforementioned *Fundamental Research Funds*, which were expanded to the higher education sector later in 2008.

Stage 3 (2014 to present): Refinement of the funding mechanism

The previous stage witnessed a rapid increase in the total amount of funds for basic research which has led to certain positive outcomes, such as the rapid expansion of the research personnel and outputs. Since 1985, the central government has been expanding the channels and volumes of projects funding for basic research. This undoubtedly increased the proportion of competition-based funding mechanisms, yet it caused a series of negative impacts, especially it resulted in a very fragmented funding system. By 2014, there were more than 100 S&T funding programs set up at the central government level. These programs were set up by multiple agents and they generally fund overlapped research projects, and thus caused huge inefficiency issues and a waste of national resources (Kang, 2007). To address this problem, in March 2014, the State Council integrated these over 100 S&T funding programs into Five Major Plans, namely NSFC, National Key R&D Programmes, National S&T Major Programmes, Technological Innovation Guiding Fund, and Bases and Talents Programme. Of these five, only NSFC specifically focuses on basic research. By 2017, the 3-years transitional period from the old funding system to the new Five-Major-Plans System was basically completed and these Five Major Plans are established as China’s current main science and technology funding schemes.

**FIGURE 3:**
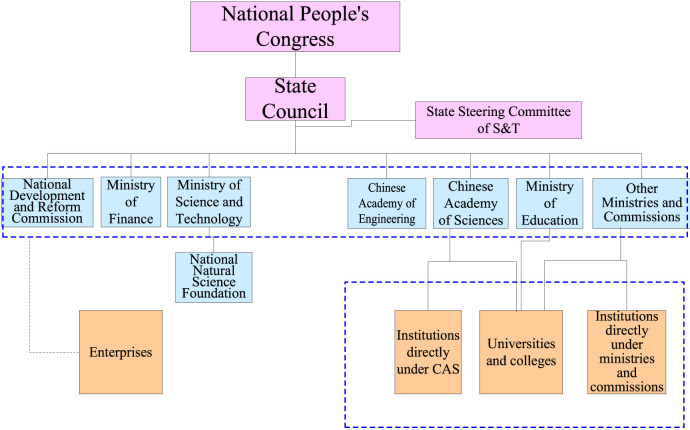
Science and technology funds management system of central government in China. Source: Author’s own.

The Ministry of Science and Technology, Ministry of Education, NSFC^5^ and Chinese Academy of Sciences are the main funding agents for China’s basic research (Figure 3). Among them, NSFC is the major funding agent in natural science, which mainly aims to encourage the curiosity driven research targeting for excellent science. Take the year 2017 as an example, its total budget was 26.7 billion Yuan (RMB), accounting for 27% of China’s total investment in basic research (Zhou and Zhao, 2019).

Along with this increasing expansion of its total investment, NSFC has witnessed a huge growth of the total amount of project applications over the years, rising from 153,800 in 2011 to 281,200 in 2020. This has enormously increased the funding agency’s workload and further raised the issues of low funding efficiency. The evaluation process in NSFC generally comprises two stages: double-blinded peer review stage and panel committee meeting review stage. In general, one project requires 5–7 experts to review in the double-blind review stage, and 281,000 applications will demand 1.4–1.9 million hours as input to fulfil the task. This has directly led to the high cost of human resources both in terms of researchers’ inputs as well as the funding agency’s inputs. Therefore, how to create the appropriate funding scheme to guide the research institutes and universities to follow their own missions has been another challenge for China’s future reform of its funding system. In 2021, the Chinese government urged to formulate “Strategic Scientific Plans and Programmes” to build the national strategic power of science and technology (*Guojia Zhanlue Keji Liliang*). Basic research capacity has been signaled as a key focus of this strategic power. However, several challenges are observed: how to restructure the funding system to set up the stabilized funding scheme for basic research at the national level; how to nurture new missions for national research institutes at the organizational level, so that they can align well with the national needs as well as to distinguish themselves from the universities.

Now, block grants are channeled to universities to support their goals of being the world’s leading research centers in certain disciplines. In 2015, the State Council set up the goal of building the world’s first-class universities and disciplines (also known as double-first-class programme) (State Coucil, 2015), in which it integrated the precedent 985 Programme and the 211 Programme. This served the purpose of optimizing the central government’s funding system for the higher education sector. However, China is still in the process of reforming its research funding system so that to level up the block grants to support national research institutes in responding to the national strategic needs. This has been evidenced by the recent speech from President Xi, who has urged China to “leverage state laboratories to enhance the national strategic power of science and technology” (Xi, 2016). This was further endorsed by the 2021 Government Work Report. The report proposed to advance the construction of state laboratories system and further to improve the national strategic S&T capacity through continuously providing more stabilized research funding schemes.

## Features of China’s Central Government Funding System for Basic Research

In this section, we aim to capture how the features of China’s central government funding system evolve for basic research. The analysis covers two aspects: drivers of basic research and features of funding recipients.

**FIGURE 4:**
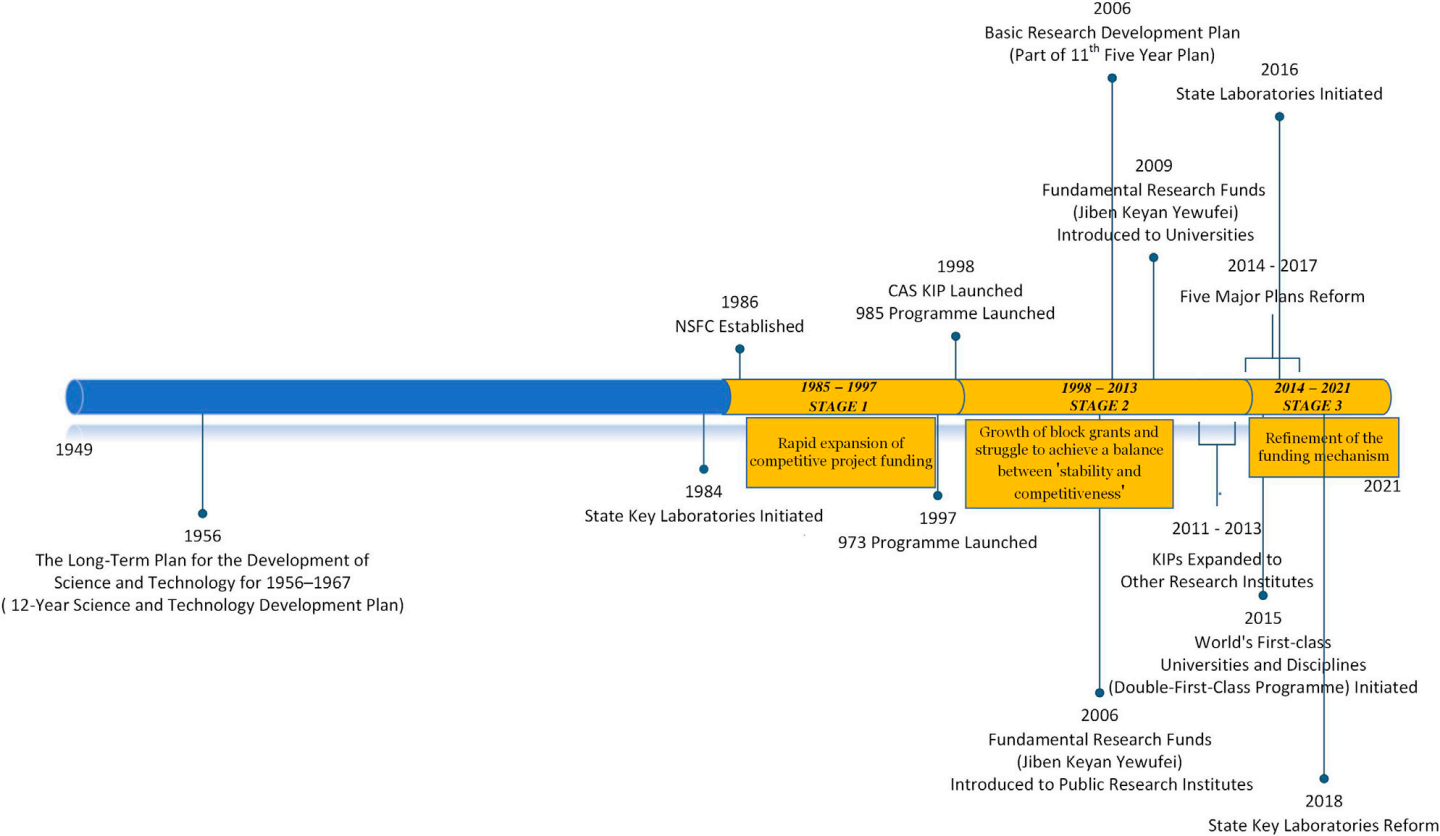
Milestones of China’s central government funding system evolution for basic research. Source: Author’s own.

In the past few decades, China has mobilized different funding channels and mechanisms to fund its basic research so as to meet its different demands at different stages of scientific development (illustrated in Figure 4). Its funding mechanisms had been shifted from a competition dominant project funding mechanism (from 1985 to 1998), represented by the establishment of NSFC and the launch of 973 Programme, toward a more mixed form of block grants and project funding (after 1998), in which block grants (represented by CAS KIP and the Fundamental Research Funds) provided stable financial support, while project funding (represented by the NSFC) offered a competitive selection based scheme to select excellent research. This shift aligned with the evolution of the country’s research system. After the science and technology research reform in 1985, the country was in the transition period from its planning economy toward a market-oriented economy, limited research resources were channeled to the high-performance research through competitions, and the emphasis in that period was to level up the national research capacities and to rejuvenate national research institutes. From 1998 onward, the country increased its block grants and a more mixed funding mechanism was adopted (project funding represented by the Five Major Plans and block grants represented by the continuous Fundamental Research Funds, Grants to the State Labs and State Key Labs as well as to the first-class universities) so that to both encourage excellent research as well as to guarantee certain national research institutes and universities for more stabilized funding in aligning with their missions and the national demands. In the recent years (since 2014), the Chinese government argued that more attention and funds need to be channeled to build the national strategic power of science and technology (represented by the recent reform in its state laboratories and key laboratories as well as to argue for “Strategic Scientific Plans and Programs”).

From the historical evolution, we observe that the Chinese central government leveraged different funding mechanisms to support basic research at different stages. Currently, the country is struggling to make a balance between block grants and project funding in the government’s basic research funding mechanism. We observe that the country’s basic research funding system has been heavily overshadowed by competition-oriented project funding scheme. In addition, suffering from the shortage in long-term oriented stabilized block grant funding scheme, the country’s current funding system cannot provide adequate incentives to encourage funding recipients to continuously devote themselves into a particular research topic. Moreover, this lack of continuity undermines the country’s capacity to make scientific breakthroughs to win for the country a Nobel Prize. It becomes obvious that purely relying on the project funding just cannot fully cater to its national demand.

However, China is faced with persistent challenges in levelling up its indigenous innovation capacity and leveraging its research for the purpose of industrial innovative activities. These challenges have been perceived as bottlenecks for the country’s further development and urgently need to be addressed, especially under the pressure of the ongoing trade war between China and the US. Basic research has been perceived as playing a crucial role in this progress. Increasing investment scales in basic research and improving its efficiency in responding to the national strategic demands will be the focal points for the country’s future policy measures. Therefore, how to reform its funding system to create incentives to improve its research qualities, instead of just focusing on the expansion of publication quantities, as well as to enable its research system to respond to the new challenges and demands has become one of the major concerns for China’s future science and technology system reform.

Another persistent challenge faced by China is the increasing homogeneity of the national research institutes and universities. We observe both the national research institutes and the universities need to apply for basic research funding through the same channel. There is no clear distinction of missions between national research institutes and research universities, which has caused over-competition between the two and further leads to waste and low efficiency of national research resources. Therefore, it has become one of the pressing issues for the country to further optimize its public funding system for the two types of funding recipients, namely national research institutes and universities.

## Conclusion

To conclude, we have offered an analytical framework to examine the central government funding system for basic research from two dimensions: (1) drivers of basic research; (2) funding recipients. Specifically, we have applied this framework into the historical analysis of China’s central government funding system for basic research since 1985. The historical evolution of China’s central government funding system for basic research has featured three distinctive stages.

There have been shared trends of recent changes in China’s basic research funding system compared with the global changes. For example, China recently put emphasis on the role of strategic basic research that aims to bring basic research to fulfil the economic and social demand. This has been the case for most OECD countries, which are dominated by programs that serve specific government missions, such as defense, agriculture, health, energy, and other activities (Mowery, 2009). For mission-oriented research, it has been argued that public investment should play a role in shaping the market, coordinating, and collaborating with private investment (Kattel and Mazzucato, 2018; Mazzucato, 2018). In this sense, how to create the crowd-in effect for public investment in basic research and build public-private partnership to fully leverage the public money to serve national demands should be a concern for China’s future policy measures. NSFC has made its effort to create joint research programs with enterprises to motive private investment for supporting basic research. However, China has only kickstarted its exploration in this journey. Furthermore, China’s recent active debates on how to motivate a wider range of different stakeholders, apart from the scientist community but also industry actors, governments, and other stakeholders, to be involved in project selection to direct basic research following social-economic objectives have also been shared in the other OECD countries. However, how to go beyond the previous peer review evaluation approach to build more efficient assessment approaches to achieve this purpose would be a huge challenge for the country.

Compared with its counterparts of leading innovative countries, China is still in its transition period to create appropriate incentive systems to efficiently mobilize its research system to target national missions. On the one hand, as having discussed in our paper, the country is currently suffering from the increasing homogeneity among the national research institutes and universities. Thus, how to nurture efficient coordination among different research organizations would be key for the country to achieve this goal. On the other hand, the Chinese enterprises devote limited proportions of their R&D spending on basic research compared to the US companies.

## Data Availability

The original contributions presented in the study are included in the article/Supplementary Material. Further inquiries can be directed to the corresponding author.

## Footnotes

1 Access at: OECD https://stats.oecd.org/Index.aspx?DataSetCode=PERS_FORD

2 Access at: NSF https://ncses.nsf.gov/pubs/nsb20206/publication-output-by-region-country-or-economy

3 Due to the lack of accessible data before 2008, here we present China’s historical data from 2008 instead of 2000. Note: The US and China adopted different measures for basic research which may cause differences when comparing their total amount of investment in basic research. To elaborate, for example, the student education investment related to basic research has been included in the US data, while excluded in the Chinese dataset (i.e., in China, the educational related programs, such as graduate students’ education, 985 and double-first programs, belong to the expenditures of the Chinese Ministry of Education, which has not been counted in this basic research dataset by Ministry of Finance)

4 Calculated by authors based on the statistical data of the funded projects from NSFC 2019 annual report

5 In 2018, NSFC was affiliated to the Ministry of Science and Technology (MOST) but kept its due independence in operation
