# Supplementary material for: Evolution and Features of China’s Central Government Funding System for Basic Research
Source: Front Res Metr Anal. 2021 Dec 23;6:751497. doi: 10.3389/frma.2021.751497 (PMC8732960; doi:10.3389/frma.2021.751497)
Supplement: Supplementary file 1 [file DataSheet1.docx]

# Appendix

Table: Data sources

|  | Types of data | | Examples | Access source data |
| --- | --- | --- | --- | --- |
| Secondary Data | Journal articles, news articles; | | |  |
| Primary Data | Policy documents | Medium and Long-term Plan for the Development of Science and Technology for 1956–1967 | | Accessed at: http://www.gov.cn/gongbao/content/2006/content_240244.htm |
|  |  | Five Major Scientific Plans Reform | | Accessed at: http://www.gov.cn/zhengce/content/2015-01/12/content_9383.htm |
|  |  | Research funding systems reforms policies and guidance, such as Chinese Fundamental Research Funds for the Central Research Institutes and for Central Universities (Jiben Keyan Yewufei) | | Accessed at: <http://www.gov.cn/xinwen/2016-07/27/content_5095236.htm>;  Accessed at: http://www.moe.gov.cn/jyb_xxgk/moe_1777/moe_1779/201610/t20161028_286795.html |
|  | Statistics | National Patterns of R&D Resources: 2018–19 （National Center for Science and Engineering Statistics, US NSF） | | Accessed at: https://ncses.nsf.gov/pubs/nsf21325#data-tables |
|  |  | China Statistical Yearbook on Science and Technology,1985-2019 | |  |
|  |  | Central Level and Local Governments' General Public Budget Expenditure,2020 | | Accessed at: <http://yss.mof.gov.cn/2020zyjs/202106/t20210629_3727246.htm>  Accessed at: <http://yss.mof.gov.cn/2020zyjs/202109/t20210917_3753567.htm> |
|  | Organisational Reports | NSFC Annual Reports,2016-2020 | | Accessed at: http://www.nsfc.gov.cn/publish/portal0/zfxxgk/04/05/ |
|  |  | CAS KIP Evaluation Report | | Chinese Academy of Sciences. 2012. The successful practices of constructing national systems of innovation with Chinese characteristic: The evaluation of Chinese Academy of Sciences’ Knowledge Innovation Programme (1998-2010). Science Press. ISBN:9787030327970 |
|  |  | China’s Basic Research Competitiveness Report,2020 | | Zhong Y. et.al. 2021. China’s Basic Research Competitiveness Report 2020. Science Press. ISBN：9787030665683 |
